# Major hemorrhage in chronic lymphocytic leukemia patients in the US Veterans Health Administration system in the pre‐ibrutinib era: Incidence and risk factors

**DOI:** 10.1002/cam4.2134

**Published:** 2019-04-14

**Authors:** Peter Georgantopoulos, Huiying Yang, LeAnn B. Norris, Charles L. Bennett

**Affiliations:** ^1^ William Jennings Bryan Dorn Veterans Affairs Medical Center Columbia South Carolina; ^2^ Southern Network on Adverse Reactions (SONAR), South Carolina Center of Economic Excellence for Medication Safety, College of Pharmacy University of South Carolina Columbia South Carolina; ^3^ Department of Epidemiology and Biostatistics, Arnold School of Public Health University of South Carolina Columbia South Carolina; ^4^ Pharmacyclics LLC, an AbbVie Company Sunnyvale California

**Keywords:** chronic lymphocytic leukemia, hemorrhage, ibrutinib

## Abstract

Chronic lymphocytic leukemia (CLL) patients are at increased risk for major hemorrhage (MH). We examined incidence of and risk factors for MH in CLL patients before introduction of newer CLL therapies such as ibrutinib, which includes bleeding risk. This study included 24 198 CLL patients treated in the VA system before FDA approval of ibrutinib as CLL therapy. Data came from VA databases from 1999 to 2013. MH incidence was 1.9/100 person‐years (95% CI: 1.8‐1.9), with cumulative incidences of 2.3%, 5.2%, and 7.3% by year 1, 3, and 5, respectively. Median time from CLL diagnosis to MH was 2.8 years (range: 0‐15.7 years). In multivariate analyses, concurrent anticoagulant and antiplatelet use (HR: 4.2; 95% CI: 3.2‐5.6), anticoagulant use only (HR: 2.6; 95% CI: 2.3‐3.1), and antiplatelet use only (HR: 1.5; 95% CI: 1.3‐1.7) increased MH risk vs not receiving those medications; being nonwhite, male, having MH history, renal impairment, anemia, thrombocytopenia, and alcohol abuse were associated with increased MH risk. These pre‐ibrutinib data are important for providing context for interpreting MH risk in ibrutinib‐treated patients. As ibrutinib clinical use is increasing, updated analyses of MH risk among ibrutinib‐treated VA patients with CLL may provide additional useful insight.

## 1 INTRODUCTION

Patients with chronic lymphocytic leukemia (CLL) are at increased risk for major hemorrhage compared with the general population.^1^ An analysis of the Surveillance, Epidemiology, and End Results (SEER)‐Medicare database showed that the hazard ratio (HR) for development of a major hemorrhage among 6717 treated CLL/small lymphocytic lymphoma (SLL) patients compared with 14 816 age‐ and gender‐matched noncancer patients was 8.3 (95% confidence interval [CI]: 7.5‐9.2).^1^ Use of anticoagulants or antiplatelet agents is common in CLL patients (25%‐54%),^2^, ^3^ who are generally elderly^4^ and often have comorbid conditions,^5^, ^6^ a number of which require use of anti‐hemostatic medications.^7^, ^8^

Ibrutinib, a first‐in‐class, once‐daily inhibitor of Bruton's tyrosine kinase (BTK), is approved in the United States for treatment of CLL/SLL.^9^ Clinical studies indicate that use of ibrutinib and other BTK inhibitors is associated with platelet dysfunction and increased bleeding risk.^10^, ^11^, ^12^, ^13^, ^14^, ^15^, ^16^, ^17^ In order to put ibrutinib into a clinical context, it is important to understand incidence of and risk factors for major hemorrhage in the CLL patient population before ibrutinib is widely used. Particularly, the effect sizes of potential risk factors (such as sociodemographic characteristics, comorbid conditions, and anticoagulant and antiplatelet medications) for major hemorrhage among CLL patients were not well established prior to introduction of ibrutinib into clinical practice.

The Veterans Health Administration (VA) medical system, the largest integrated healthcare delivery system in the United States, with over 140 VA medical centers, approximately 9 million veteran enrollees, and medical service provision to over 5 million patients annually, is a particularly important system to describe incidence of and risk factors for major hemorrhage among CLL patients, given the large CLL patient population among VA patients and high rates of comorbid illnesses among these patients.^18^, ^19^ VA electronic databases date back to the 1990s and include detailed longitudinal data on medical diagnoses, surgical procedures, use of all pharmaceuticals administered by the VA, hospitalization, cancer diagnoses, and vital status for Veterans who receive care in the VA medical system.

The objective of this study was to determine the incidence of and risk factors for major hemorrhage in a cohort of veterans with newly diagnosed CLL who received care in the VA prior to introduction of ibrutinib into the VA medical system.

## 2 METHODS

### 2.1 Data sources

We conducted a retrospective cohort study, including veterans with CLL treated in VA medical centers from January 1, 1999, to December 31, 2013. Data were obtained from the VA, and the following datasets were used: VA MedSAS inpatient and outpatient patient care datasets, Master Vital Status Dataset, Mini Vital Status Dataset, and the VA Pharmacy Benefits Management Inpatient and Outpatient datasets. The VA Master Vital Status and VA Mini Vital Status datasets provide patient‐level information, while VA MedSAS patient care datasets provide visit‐level information.^20^

### 2.2 Patients and variables

MedSAS files were queried for VA persons with CLL using the ICD9 code of 204.1x. Patients were eligible if they had a diagnosis of CLL recorded at ≥2 visits with >30 days’ gap between the visits and were ≥18 years old. In order to define a newly diagnosed patient cohort, only those patients who had at least one VA visit ≥6 months prior to the first CLL diagnosis were included. Follow‐up was from initial CLL diagnosis until death, drop out from the VA system, the data cut‐off date (December 31, 2013), or onset of major hemorrhage, whichever was the earliest. The primary outcome was major hemorrhage as defined using the framework described in Schulman et al 2005.^21^ Hemorrhages treated with blood transfusion within 7 days of occurrence or hemorrhage occurring in the central nervous system regardless of blood transfusion were defined as major hemorrhage (detailed codes listed in Supplementary Table S1). Only first occurrences of a major hemorrhage for individual patients were included in the analysis. Patients with a major hemorrhage occurring after the first CLL diagnosis were classified as having the outcome of interest.

Potential risk factors for major hemorrhage^22^ that were evaluated included race, gender, age at the time of CLL diagnosis in the VA, and relevant medical histories (major hemorrhage, anemia, thrombocytopenia, hypertension, ischemic stroke, atrial fibrillation, coronary artery disease, hepatic disease, renal impairment, neurological diseases, and alcohol abuse) recorded during 6 months prior to the first CLL diagnosis. All medical histories were determined by using ICD9 codes (detailed codes listed in Supplementary Table S1).

Antiplatelet and anticoagulant medications (see Supplementary Table S2 for list of medications) were the main risk factors of interest in this study. Use of antiplatelet drugs and/or anticoagulants was defined and analyzed in three different methods: (a) Anytime use (yes/no): defined as having used antiplatelet and/or anticoagulant drugs if patients received antiplatelet and/or anticoagulant drugs anytime during the observational follow‐up period. (b) Recent use (yes/no): defined as having used antiplatelet and/or anticoagulant drugs during 90 days prior to the onset of major hemorrhage for those with major hemorrhage. For those without major hemorrhage, each of three 90‐day periods was evaluated (90 days post CLL diagnosis, 90‐day mid‐point of the observation period, and 90 days prior to the end of observation period). (c) Time‐varying covariate: timing and duration of antiplatelet and/or anticoagulant use were considered in a Cox‐proportional hazards model where they were analyzed as a time‐dependent repeated covariate measurement.

### 2.3 Analyses

The incidence of major hemorrhage among CLL patients was calculated as the number of major hemorrhages divided by the total number of person‐years during the study timeframe. The cumulative incidence function for major hemorrhage was estimated using a subdistribution model^23^ where death was considered a competing risk. Univariate analysis was conducted for individual risk factors, and relative risk (RR) and 95% CI of developing major hemorrhage were calculated for all potential risk factors. A multivariate Cox‐proportional hazards regression analysis^24^ was conducted to determine independent risk factors and estimate hazard ratios for major hemorrhage using a backward stepwise approach. A time‐dependent covariate for antiplatelet and anticoagulant use was incorporated into the multivariate model with four mutually exclusive groups (no anticoagulant or antiplatelet use, antiplatelet use only, anticoagulant use only, and simultaneous use of both) to estimate the degree of association to major hemorrhage in order to account for the intermittent use of those medications.

All analyses were conducted using SAS Enterprise Guide. The Institutional Review Board at the WJB Dorn Veterans Health Administration Medical Center approved the study protocol.

## 3 RESULTS

**Figure 1:**
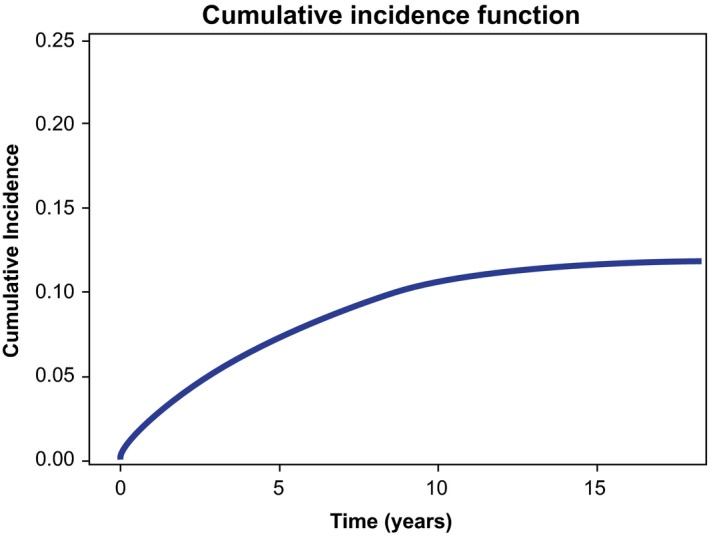
Cumulative incidence function of major hemorrhage in patients with CLL

Between 1999 and 2013, a total of 24 198 patients with CLL received care in VA medical centers and were included in the analysis. The median duration of follow‐up after CLL diagnosis was 4.1 years (range: 0‐18.2 years). Overall, 2 207 patients (9.1%) developed a major hemorrhage after a CLL diagnosis during the follow‐up time‐period. The median time from CLL diagnosis to major hemorrhage was 2.8 years (range: 0‐15.7 years). The incidence rate of major hemorrhage was 1.9 per 100 person‐years (95% CI: 1.8‐1.9 per 100 person‐years). Cumulative incidence rates of major hemorrhage, after taking death as competing risk into consideration, by years 1, 2, 3, 4, 5, and 10 were 2.3%, 3.8%, 5.2%, 6.3%, 7.3%, and 10.6%, respectively (Figure 1).

The majority of patients were male (98%), white (85%), and 65 years or older (71%). Hypertension and coronary artery disease were common comorbid illnesses in these patients (57% and 23%, respectively). Atrial fibrillation was recorded in 7% of these patients. Anemia and thrombocytopenia were diagnosed in 13% and 5% of patients, respectively. Other potential risk factors, including hepatic disease, renal impairment, neurological diseases, alcohol abuse, and ischemic stroke, were recorded in 1.4% to 10% of patients. History of major hemorrhage had occurred in 0.3% of these patients during 6 months prior to their CLL diagnosis (Table 1).

**Table 1:** VA CLL patient characteristics at baseline and univariate analysis results

	Total CLL cohort (N = 24 198)	MH (n = 2207)	No MH (n = 21 991)	Univariate analysis RR (95% CI)	*P* value
Demographic characteristics, n (%)					
Male	23 798 (98)	2184 (99)	21 614 (98)	1.6 (1.1, 2.4)	0.02
Race					
White	20 503 (85)	1739 (79)	18 764 (85)	Reference	
Black	1868 (8)	259 (12)	1609 (7)	1.6 (1.4, 1.8)	<0.0001
Other	1827 (8)	209 (10)	1618 (7)	1.3 (1.2, 1.5)	<0.0001
Age at diagnosis, n (%)					
<65	7073 (29)	728 (33)	6345 (29)	Reference	
≥65 ‐ <75	7433 (31)	662 (30)	6771 (31)	0.9 (0.8, 1.0)	0.005
≥75	9692 (40)	817 (37)	8875 (40)	0.8 (0.7, 0.9)	<0.0001
Medical history during 6 months prior to CLL diagnosis, n (%) (those without corresponding medical history as reference group)					
Major hemorrhage	73 (0.3)	28 (1)	45 (0.2)	4.2 (3.2, 5.7)	<0.0001
Anemia	3116 (13)	462 (21)	2654 (12)	1.8 (1.6, 2.0)	<0.0001
Hepatic diseases	333 (1)	44 (2)	289 (1)	1.5 (1.1, 1.9)	0.009
Renal impairment	2412 (10)	303 (14)	2109 (10)	1.4 (1.3, 1.6)	<0.0001
Thrombocytopenia	1095 (5)	141 (6)	954 (4)	1.4 (1.2, 1.7)	<0.0001
Alcohol abuse	721 (3)	90 (4)	631 (3)	1.4 (1.1, 1.7)	0.002
CAD	5488 (23)	562 (25)	4926 (22)	1.2 (1.1, 1.3)	0.001
Atrial fibrillation	1762 (7)	187 (8)	1575 (7)	1.2 (1.0, 1.4)	0.02
Neurological diseases	783 (3)	75 (3)	708 (3)	1.1 (0.8, 1.3)	0.7
Ischemic stroke	744 (3)	77 (3)	667 (3)	1.1 (0.9, 1.4)	0.2
Hypertension	13 726 (57)	1280 (58)	12 446 (57)	1.1 (1.0, 1.1)	0.2

CAD, coronary artery disease; CLL, chronic lymphocytic leukemia; MH, major hemorrhage; VA, Veterans Health Administration.

Univariate analysis showed that race, gender, age, history of major hemorrhage, hepatic disease, renal impairment, anemia, thrombocytopenia, coronary artery disease, atrial fibrillation, and alcohol abuse were significantly associated with major hemorrhage, but most risk factors showed moderate association (RR < 2), except history of major hemorrhage during 6 months prior to the CLL diagnosis (RR: 4.2; 95% CI: 3.2‐5.7) (Table 1).

Antiplatelet or anticoagulant medications were taken by 60% of the CLL patients during the follow‐up period; relatively more patients received antiplatelet agents (46%) than anticoagulants (34%), including 20% who used both classes of medications (Table 2). Among patients with major hemorrhage, 32% used anticoagulants and/or antiplatelets during 90 days prior to the onset of major hemorrhage, while approximately 20% of those without major hemorrhage used anticoagulants and/or antiplatelets during the 90 days either post CLL diagnosis, at the middle point of follow‐up (shown in Table 2), or prior to the end of follow‐up. Such recent use of anticoagulants and/or antiplatelets was associated with major hemorrhage with an RR of 1.8 (95% CI: 1.7‐2.0). Compared to those without use of anticoagulants or antiplatelets during the specified 90 days, use of antiplatelets only, anticoagulants only, and both anticoagulants and antiplatelets all were significantly associated with major hemorrhage, with RRs of 1.3 (95% CI: 1.1‐1.4), 2.6 (2.3‐2.9), and 4.4 (3.7‐5.3), respectively.

**Table 2:** Use of anticoagulant/antiplatelet agents in VA CLL patients during follow‐up period

	**Total CLL cohort (N = 24 198)**	**MH (n = 2207)**	**No MH (n = 21 991)**	**Univariate analysis RR (95% CI)**	***P*** **value**
Use of anticoagulant/antiplatelet agents anytime during follow‐up period, n (%)					
Use of AC (vs no use of AC)	8205 (34)	865 (39)	7340 (33)	1.3 (1.2, 1.4)	<0.0001
Use of AP (vs no use of AP)	11 149 (46)	1202 (55)	9947 (45)	1.4 (1.3, 1.5)	<0.0001
	
No use of AC or AP	9697 (40)	706 (32)	8991 (41)	Reference	
Use of AC and/or AP	14 501 (60)	1501 (68)	13 000 (59)	1.4 (1.3, 1.5)	<0.0001
Use of AP only	6296 (26)	636 (29)	5660 (26)	1.4 (1.3, 1.5)	<0.0001
Use of AC only	3352 (14)	299 (14)	3053 (14)	1.2 (1.1, 1.4)	0.002
Use of both AC and AP^a^	4853 (20)	566 (26)	4287 (20)	1.6 (1.4, 1.8)	<0.0001
					
Recent 90‐day use of anticoagulant/antiplatelet agents^b^, n (%)					
Use of AC (vs no use of AC)	1765 (7)	393 (18)	1372 (6)	2.8 (2.5, 3.0)	<0.0001
Use of AP (vs no use of AP)	3466 (14)	405 (18)	3061 (14)	1.3 (1.2, 1.5)	<0.0001

No use of AC or AP	19 213 (79)	1493 (68)	17 720 (81)	Reference	
Use of AC and/or AP	4985 (21)	714 (32)	4271 (19)	1.8 (1.7, 2.0)	<0.0001
Use of AP only	3220 (13)	321 (15)	2899 (13)	1.3 (1.1, 1.4)	<0.0001
Use of AC only	1519 (6)	309 (14)	1210 (6)	2.6 (2.3, 2.9)	<0.0001
Use of both AC and AP^a^	246 (1)	84 (4)	162 (1)	4.4 (3.7, 5.3)	<0.0001

AC, anticoagulant; AP, antiplatelet.

a Use of AC and/or AP anytime during the follow‐up period. For those using both AC and AP, their use may not be at the same time.

b Use of AC and/or AP 90 days prior to MH onset for those with MH or during the middle 90 days of the follow‐up period for those without MH.

Table 3 summarizes variables retained in the final model from the multivariable analysis. Demographically, being male (HR: 2.0; 95% CI: 1.3‐3.1) and, as compared to white, being black (HR: 1.6; 95% CI: 1.4‐1.8) or other (HR: 1.8; 95% CI: 1.5‐2.1) were associated with increased risk for major hemorrhage after CLL diagnosis. Among history of medical conditions, having a major hemorrhage 6 months prior to CLL diagnosis was most strongly associated with the risk of developing a major hemorrhage after CLL diagnosis (HR: 2.8; 95% CI: 1.8‐4.4). Other medical histories that were moderately associated with developing a major hemorrhage after CLL diagnosis in the final model were renal disease (HR: 1.4; 95% CI: 1.2‐1.6), anemia (HR: 2.0; 95% CI: 1.8‐2.2), thrombocytopenia (HR: 1.3; 95% CI: 1.1‐1.6), and alcohol abuse (HR: 1.3; 95% CI: 1.0‐1.7).

**Table 3:** Final multivariate, time‐dependent Cox proportional hazard model determining the risk factors for and estimating the hazard ratios for developing a major hemorrhage among chronic lymphocytic leukemia patients^a^

Risk factor retained in the final model	Hazard ratio (95% Confidence Interval)
Male	2.0 (1.3, 3.1)
Race	
White	Reference
Black	1.6 (1.4, 1.8)
Other	1.8 (1.5, 2.1)
Medical history (6 months prior to CLL diagnosis)	
Major hemorrhage	2.8 (1.8, 4.4)
Renal impairment	1.4 (1.2, 1.6)
Anemia	2.0 (1.8, 2.2)
Thrombocytopenia	1.3 (1.1, 1.6)
Alcohol abuse	1.3 (1.0, 1.7)
Use of anticoagulant and antiplatelet	
Neither anticoagulant or antiplatelet use	Reference
Anticoagulant and antiplatelet use	4.2 (3.2, 5.6)
Anticoagulant use only	2.6 (2.3, 3.1)
Antiplatelet use only	1.5 (1.3, 1.7)

a CLL: chronic lymphocytic leukemia. Not retained: these variables did not reach statistical significance after adjusting for other variables. Age at CLL diagnosis; medical history of hypertension, hepatic diseases, ischemic stroke, coronary artery disease, atrial fibrillation, and neurological diseases were not retained in any model.

Use of either anticoagulant or antiplatelet medications significantly increased the risk for major hemorrhage. The simultaneous use of anticoagulant and antiplatelet medication had the strongest association with developing a major hemorrhage (HR: 4.2; 95% CI: 3.2‐5.6). Use of only anticoagulant medication (HR: 2.6; 95% CI: 2.3‐3.1) or use of only antiplatelet medication (HR: 1.5; 95% CI: 1.3‐1.7) was also independently associated with risk of major hemorrhage.

## 4 DISCUSSION

In this large retrospective study of newly diagnosed CLL patients who received care between 1999 and 2013 (ie, the pre‐ibrutinib era) in the VA medical system, we observed an average incidence rate of 1.9 major hemorrhages per 100 person‐years post CLL diagnosis. Interestingly, the risk of major hemorrhage was not constant, with higher risk in earlier years as exhibited by a nonlinear cumulative incidence function with a plateau (Figure 1). Cumulative incidence rates by year 1, year 3, and year 5, after taking death as a competing risk into consideration, were 2.3%, 5.2%, and 7.3%, respectively.

In an older Medicare CLL patient population who received cancer therapy from 2000 to 2011, incidence of major hemorrhage after cancer treatment was 6.0 per 100 person‐years compared to 1.6 per 100 person‐years in an age‐ and gender‐matched noncancer control group.^1^ The approximately threefold higher incidence of major hemorrhage in the Medicare CLL patients compared to the current VA CLL patients could in part be explained by the relatively older population of the Medicare patients and differences in the time frame for calculating incidence (calculated from time of initiating treatment rather than time from first CLL diagnosis).

The incidence of major hemorrhage in ibrutinib‐treated patients with CLL and other B‐cell malignancies varies greatly in the literature, likely due to differences in the patient population, treatment and follow‐up duration, and definition of major hemorrhage. In a pooled analysis of four randomized trials in patients with CLL or mantle cell lymphoma, the incidence of major hemorrhage was similar (3.2 vs 3.1 per 1000 person‐months) between ibrutinib‐ and comparator‐treated patients.^25^ In the real‐world setting, reported incidence of major hemorrhage ranged from 1.2% (N = 165)^26^ to 19% (N = 70)^27^ of patients treated with ibrutinib; in the largest study conducted to date, by Pavlik and colleagues (N = 437), major hemorrhage was reported in 3.2% of ibrutinib‐treated patients.^28^

In our study, the risk factors with strong associations (HR > 2.5) with major hemorrhage among these CLL patients included history of major hemorrhage and concomitant use of anticoagulants and antiplatelet agents. Major hemorrhage risks were 4.2‐fold higher among patients who received both anticoagulants and antiplatelets simultaneously, 2.6‐fold higher with anticoagulants alone, and 1.5‐fold higher with antiplatelet agents compared to those with no use of either anticoagulants or antiplatelets. These results showed that (a) the effect of anticoagulants on the risk of major hemorrhage is significantly greater than use of antiplatelets, and (b) there is an additive effect of using both anticoagulants and antiplatelets on the risk of major hemorrhage. Therefore, caution is advised in the use of both anticoagulants and antiplatelets simultaneously to minimize the risk of major hemorrhage. In addition, other medical histories that were moderately associated with developing major hemorrhage after CLL diagnosis were renal disease, anemia, thrombocytopenia, and alcohol abuse.

Risk factors for major bleeding are well established in patients with atrial fibrillation requiring anticoagulation therapy. Several risk scores (HAS‐BLED Score, ATRIA Bleeding Score, HEMORR_2_HAGES Score, CHA_2_DS_2_‐VASc Score) have been developed to predict major bleeding risk in anticoagulated patients with atrial fibrillation.^29^ Risk factors for major hemorrhage identified in the current VA CLL patients were the components of these risk scores. However, in these risk scores, the risk quantification for anticoagulant and antiplatelet treatment was not differentiated.

In a clinical study of 4060 patients with atrial fibrillation receiving anticoagulation therapy, major hemorrhage was reported in 9.2% of the patients during an average 3.5 years of follow‐up,^30^ which appears to be higher than what we observed in the VA CLL patients with or without anticoagulation therapy (ie, 5.2% cumulative incidence by year 3). In the multivariate analysis, in addition to age, history of congestive heart failure, diabetes, and hepatic or renal disease, use of warfarin and aspirin was significantly associated with risk of major hemorrhage (HR: 1.8 and 2.0, respectively).^30^ A similar degree of association with anticoagulants and antiplatelets was observed in the current VA CLL patients (HR: 2.6 and 1.5, respectively). Furthermore, among Medicare patients with atrial fibrillation who were receiving warfarin, incidences of intracranial hemorrhage and major extracranial hemorrhage were reported to be 8.6 and 25.9 per 1000 person‐years, respectively, while for patients who were receiving novel oral anticoagulant therapy (ie, dabigatran, rivaroxaban, or apixaban), major hemorrhage incidence rates were reported as ranging from 3.3 to 5.6 per 1000 person‐years for intracranial bleeding and 14.6 to 35.5 per 1000 person‐years for major extracranial bleeding.^31^

The basic pathophysiology of hemorrhage in CLL patients is relevant to consider. Bleeding in CLL patients may result from immune‐mediated thrombocytopenia, marrow underproduction of platelets, thrombocytopenia from splenic sequestration, impaired collagen‐mediated platelet aggregation, or other factors.^32^, ^33^ These pathophysiological factors are not typically observed in other chronic leukemias, such as in the chronic phase of chronic myelogenous leukemia.^34^, ^35^

Our study has limitations. While our study is one of the largest to date reporting real‐world major hemorrhage experiences among persons with CLL in the pre‐ibrutinib era, our dataset represented a unique veteran population and did not include large numbers of women. Thus, findings from the VA medical setting may not be fully extrapolatable to the general US population. In addition, some veterans may receive CLL care outside of the VA, and this information may not be captured in our VA databases; however, the likelihood that a veteran enrolled in the VA system would receive care outside the VA system is minimal, given how much the VA alleviates patients’ financial burdens to healthcare. Finally, all medical conditions were defined by using ICD9 codes. Hence, limitations typically associated with use of electronic medical records in an observational study are present.^36^ An additional limitation is that our study does not contain a matched control group of VA patients without CLL; thus, we could not quantify relative risk increase in major hemorrhage because of CLL in the VA population.

We conclude that among VA patients with CLL in the pre‐ibrutinib era, cumulative incidence rates of major hemorrhage were 2.3%, 5.2%, and 7.3% by year 1, 3, and 5 post CLL diagnosis, respectively. Prior history of major hemorrhage and concomitant use of anticoagulants and antiplatelets are strongly associated with the risk of major hemorrhage. These pre‐ibrutinib data are important baseline information to provide a context for interpretation of major hemorrhage risk in ibrutinib‐treated patients. As the use of ibrutinib in real‐world settings is increasing, updated analyses of major hemorrhage risk among ibrutinib‐treated VA patients with CLL may provide additional insight to optimize the management of CLL patients.

## 5 AUTHORSHIP CONTRIBUTIONS

PG: design of the study, data analysis and interpretation, drafting and critical revision of the manuscript; HY: design of the study, data interpretation, drafting and critical revision of the manuscript; LBN: design of the study, identification of antiplatelet and anticoagulant drugs, data interpretation, critical review of the manuscript; CLB: design of the study, data interpretation, drafting and critical revision of the manuscript.

## 6 ROLE OF THE FUNDER/SPONSOR

Pharmacyclics LLC, an AbbVie Company, sponsored part of the study. Study investigators collected and analyzed the data, and drafted/provided critical review of the manuscript. Medical writing support was funded by the sponsor.
