# Supplementary material for: Major hemorrhage in chronic lymphocytic leukemia patients in the US Veterans Health Administration system in the pre‐ibrutinib era: Incidence and risk factors
Source: Cancer Med. 2019 Apr 14;8(5):2233–40. doi: 10.1002/cam4.2134 (PMC6536934; doi:10.1002/cam4.2134)
Supplement: Supplementary file 1 [file CAM4-8-2233-s001.docx]

Supplementary Table 1: International Classification of Diseases, Ninth Revision, Clinical Modification (ICD-9-CM) Codes Used to Define Relevant Medical Conditions.

| Medical Condition | ICD-9-CM code |
| --- | --- |
| Chronic lymphocytic leukemia | 204.1x |
| Major Hemorrhage not requiring Transfusion | 246.3, 360.43, 362.43, 362.81, 363.61, 363.62, 363.72, 376.32, 377.42, 379.23, 423.0, 430, 431, 432, 432.x, 800.10, 800.11, 800.12, 800.13, 800.14, 800.15, 800.16, 800.17, 800.2x, 800.3x, 800.6x, 800.7x, 800.80, 800.81, 800.82, 800.84, 800.85, 801.1x, 801.2x, 801.3x, 801.6x, 801.7x, 801.8x, 803.1x, 803.2x, 803.3x, 803.6x, 803.7x, 803.8x, 804.1x, 804.2x, 804.32, 804.33, 804.34, 804.35, 804.36, 804.6x, 804.7x, 804.8x, 851.0x, 851.1x, 851.4x, 851.8x, 852, 852.0x, 852.1x, 852.23, 852.24, 852.25, 852.26, 852.29, 852.3x, 852.4x, 852.5x, 853.0x, 853.1x, , 860.2, 860.3, 860.4, 860.5, 861.01, 861.11, 861.21, 861.31, 864.01, 864.11, 865.01, 865.11, 866.01, and 866.11 |
| Major Hemorrhage requiring Transfusion* | 285.1, 285.10, 286.5x, 287.9, 456.0, 456.20, 459.0, 530.21, 530.7, 530.82, 531.0x, 531.2x, 531.4x, 531.6x, 532.0x, 532.2x, 532.4x, 532.6x, 533.0x, 5332.x, 533.4x, 533.6x, 534.0x, 534.2x, 534.4x, 534.6x, 535.01, 535.11, 535.21, 535.31, 535.41, 535.51, 535.61, 535.71, 562.02, 562.03, 562.12, 562.13, 568.81, 569.3, 569.85, 578.0x, 596.7, 599.7x, 719.1x, 784.8, 786.30, 786.39, 958.2, 998.10, and 998.11 |
| Hepatic Disease | 570, 571.xx, 572.xx, and 573.x |
| Renal Disease | 250.4x, 403.xx, 404.xx, 458,21, 580.xx, 581.xx, 582.xx, 583.xx, 584.x, 585.x, 586, 587, 588.xx, 593.6, 593.7x, 593.81, 593.9, 599.7x, 791.0, 996.73, 996.81, V42.0, V45.1, and V56.xx |
| Alcohol Abuse | 291.xx, 303.xx, 305, 305.0x, 357.5, 425.5, 535.3x, 571.0, 571.1, 571.2, 571.3, and E86.00, |
| Hypertension | 401.x, 402.xx, 403.xx, 404.xx, and 405.xx |
| Anemia | 280.x, 281.x, 282, 282.xx, 283.xx, 284.xx, and 285.xx |
| Neurological Disorders | 290.xx, 331.0, 331.1x, 331.2, 331.3, 331.4, 331.5, 331.7, 331.8x, 331.9, 332.xx, 345.xx, 335.xx, 340, and 780.3x |
| Stroke | 434.xx, 435.x, and 436 |
| Atrial Fibrillation | 427.3x |
| Thrombocytopenia | 287.3x, 287.4x, and 287.5 |
| Coronary Artery Disease | 410.xx, 411.xx, 412, 413.x, 414.xx, and 429.2 |

*ICD-9 Procedure Codes used for Transfusion: 99.00, 99.03, 99.04, and 99.05. Current Procedural Terminology (CPT) code used for Transfusion: 36430

Supplementary Table 2: Antiplatelet and Anticoagulant Medications

| Antiplatelet drugs | aspirin, triflusal, clopidogrel, prasugrel, ticagrelor, ticlopidine, cilostazol, vorapaxar, abciximab, eptifibatide, tirofiban, dipyridamole, thromboxane synthase inhibitors, and terutroban |
| --- | --- |
| Anticoagulant drugs | warfarin, acenocoumarol, phenprocoumon, atromentin, brodifacoum, phenindione, heparin, low molecular weight heparin, fondaparinux, idraparinux, rivaroxaban, apixaban, edoxaban, betrixaban, darexaban, hirudin, lepirudin, bivalirudin, argatroban, dabigatran, ximelagatran, and antithrombin |
